# Immune response and safety to inactivated COVID-19 vaccine: a comparison between people living with HIV and HIV-naive individuals

**DOI:** 10.1186/s12981-022-00459-y

**Published:** 2022-07-05

**Authors:** Shi Zou, Mengmeng Wu, Fangzhao Ming, Songjie Wu, Wei Guo, Gifty Marley, Zhongyuan Xing, Zhiyue Zhang, Minxia Zeng, Chao Sun, Jianfeng Zhang, Weiming Tang, Ke Liang

**Affiliations:** 1 Department of Infectious Diseases, Zhongnan Hospital of Wuhan University, Wuhan, China; 2 Wuchang District Center for Disease Control and Prevention, Wuhan, China; 3 Department of Nosocomial Infection Management, Zhongnan Hospital of Wuhan University, Wuhan, China; 4 Department of Pathology, Zhongnan Hospital of Wuhan University, Wuhan, China; 5 Department of Pathology, School of Basic Medical Sciences, Wuhan University, Wuhan, China; 6 School of Public Health, Nanjing Medical University, Nanjing, China; 7 Wuhan University School of Basic Medical Sciences, Wuhan, China; 8 Zhuhai Livzon Diagnostics CO, LTD, Zuhai, China; 9 Provincial People’s Hospital, Guangzhou, China; 10 The University of North Carolina at Chapel Hill Project-China, Guangzhou, China; 11 Wuhan Research Center for Infectious Diseases and Cancer, Chinese Academy of Medical Sciences, Wuhan, China; 12 Hubei Engineering Center for Infectious Disease Prevention, Control and Treatment, Wuhan, China; 13 Dermatology Hospital, Southern Medical University, Guangzhou, 510095 China

**Keywords:** Inactivated COVID-19 vaccine, PLWH, Humoral immune response, Antibody neutralization

## Abstract

**Background:**

Multi-types COVID-19 vaccines have shown safety and efficacy against COVID-19 in adults. Although current guidelines encourage people living with HIV (PLWH) to take COVID-19 vaccines, whether their immune response to COVID-19 vaccines is distinct from HIV-free individuals is still unclear.

**Methods:**

Between March to June 2021, 48 PLWH and 40 HNC, aged 18 to 59 years, were enrolled in the study in Wuchang district of Wuhan city. All of them received inactivated COVID-19 vaccine (Sinopharm, WIBP-CorV, Wuhan Institute of Biological Products Co. Ltd) at day 0 and the second dose at day 28. The primary safety outcome was the combined adverse reactions within 7 days after each injection. The primary immunogenicity outcomes were SARS-CoV-2 neutralizing antibodies (nAbs) responses by chemiluminescence and total specific IgM and IgG antibodies responses by ELISA and colloidal gold at baseline (day 0), day 14, day 28, day 42, and day 70.

**Results:**

In total, the study included 46 PLWH and 38 HNC who finished 70 days’ follow-up. The frequency of adverse reactions to the first and second dose was not different between PLWH (30% and 11%) vs. HNC (32% and 24%). NAbs responses among PLWH peaked at day 70, while among HNC peaked at day 42. At day 42, the geometric mean concentration (GMC) and seroconversion rate of nAbs among PLWH were 4.46 binding antibody units (BAU)/mL (95% CI 3.18–5.87) and 26% (95% CI 14–41), which were lower than that among HNC [GMC (18.28 BAU/mL, 95% CI 10.33–32.33), seroconversion rate (63%, 95% CI 44–79)]. IgG responses among both PLWH and HNC peaked at day 70. At day 70, the geometric mean ELISA units (GMEU) and seroconversion rate of IgG among PLWH were 0.193 ELISA units (EU)/mL (95% CI 0.119–0.313) and 51% (95% CI 34–69), which was lower than that among HNC [GMEU (0.379 EU/mL, 95% CI 0.224–0.653), seroconversion rate (86%, 95% CI 64–97)]. There were no serious adverse events.

**Conclusions:**

Early humoral immune response to the inactivated COVID-19 vaccine was weaker and delayed among the PLWH population than that among HNC. This observation remained consistent regardless of a high CD4 count with effective antiretroviral therapy.

**Supplementary Information:**

The online version contains supplementary material available at 10.1186/s12981-022-00459-y.

## Background

Coronavirus disease 2019 (COVID-19) caused by Severe Acute Respiratory Syndrome Coronavirus 2 (SARS-CoV-2) has infected more than 196 million individuals and caused more than 4.2 million deaths worldwide by Jun 2021 [1, 2], posing unprecedented healthcare challenges around the world. Studies have shown that PLWH might be at an increased risk of SARS-CoV-2 infection or COVID-19 mortality, especially those with a longer duration of HIV infection, comorbidities, lower CD4^+^ T lymphocyte count (CD4 count), or unsuppressed HIV viral load (HIV-VL) [3–5]. Even with suppressive antiretroviral therapy (ART), HIV infection may still impact their immune response to COVID-19 vaccination and may influence the effect of the vaccine.

Currently, multi-types of COVID-19 vaccines have shown safety and efficacy against COVID-19 in adults [6–8]. Rapid humoral responses against SARS-CoV-2 were noted after inoculation of inactivated SARS-CoV-2 vaccine BBIBP-CorV (Beijing Bio-Institute of Biological Products—Coronaviruses) and 100% seroconversion was found in all participants on day 42 in phase 1/2 trial [9]. Similarly, seroconversion was noted in 97.6% participants after receiving inactivated vaccine WIV04 strain in phase 2 trial [10]. The United Nations AIDS program (UNAIDS) suggested that PLWH should be given priority in COVID-19 vaccinations regardless of CD4 count and HIV-VL levels [11–13]. In China, HIV infection was once listed as a contradiction for COVID-19 vaccination while the later national technical guideline encourages PLWH to take inactivated vaccines or recombinant subunit vaccines [14]. However, former studies have shown that residual inflammation on ART and ongoing immune dysregulation among PLWH may influence responsiveness to vaccination [15, 16]. Thus, further studies are essential in understanding whether PLWH has a distinct immune response to the COVID-19 vaccine compared to HIV-negative health controls (HNC).

This study aims to observe and compare the early immune response after COVID-19 vaccination (within 70 days after inoculation) between PLWH and HNC.

## Methods

### Study participants

This prospective study was performed from March to June 2021. Overall, the study enrolled 48 PLWH and 40 HNC, aged 18 to 59 years. All participants without a history of SARS-CoV-2 infection (via serological and nucleic acid test) were received inactivated COVID-19 vaccine (Sinopharm, WIBP-CorV, Wuhan Institute of Biological Products Co. Ltd) in Wuchang district of Wuhan city at day 0 and day 28 by intramuscular injection and provided written informed consent before enrollment in the trial. 46 PLWH and 38 HNC completed immunizations with inactivated COVID-19 vaccine at respective community hospitals and scheduled visits within the prescribed time. Blood samples were collected at baseline (day 0), day 14, day 28, day 42, and day 70. Clinical and laboratory data regarding the HIV status of PLWH were obtained from the China National HIV/AIDS Comprehensive Response Information Management System (CRIMS). Suppressed HIV viral load was defined as HIV viral load < 50 copies/mL. The CD4 count of the PLWH and HNC were tested with the blood samples at baseline.

### Safety assessments

Participants were required to record any solicited local and systemic reactogenicity on diary cards within 7 days of each injection. These recordings were summed and considered as the primary safety outcome [17]. Any other unsolicited symptoms recorded within 28 days after each shot served as the secondary safety outcome.

### Immunogenicity assessments

The primary humoral immunogenicity outcomes included the nAbs and the specific IgM and IgG-binding antibody responses to SARS-CoV-2, measured at baseline (day 0), day 14, day 28, day 42, and day 70. An in-house SARS-CoV-2 nAbs assay kit by surrogate virus neutralization test (Zhuhai Livzon Diagnostics Inc, Zhuhai, China) was used to determine the serum levels of nAbs against the spike protein receptor-binding domain (RBD) according to the manufacturers’ instructions. In brief, SARS-CoV-2 surrogate virus neutralization test detects total immunodominant neutralizing antibodies targeting the viral spike (S) protein receptor-binding domain in an isotype- and species-independent manner. This simple and rapid test is based on antibody-mediated blockage of the interaction between the angiotensin-converting enzyme 2 (ACE2) receptor protein and the receptor-binding domain [18]. The positive response of nAbs was defined as ≥ 10 BAU/mL. The semi-quantitative of total specific IgM and IgG antibodies were detected using an in-house-developed ELISA kit (Livzon), which used the recombinant nucleocapsid (N) and RBD antigen of SARS-CoV-2 as coating antigen. Positive responses of IgM and IgG were defined as ≥ 0.15 EU/mL and 0.18 EU/mL, respectively. The qualitative of total specific IgM or IgG antibodies were detected using an in-house-developed colloidal gold kit (Livzon). We defined seroconversion of antibodies as a change from baseline seronegative to seropositive.

### Statistical analysis

Data from all participants before or after injections were included in the immunogenicity analysis conducted. Missing values were imputed using the last observation carried forward method. The difference between groups was examined by Mann–Whitney U test. For categorical variables, n (%) was used for description and was examined by Chi-square test or Fisher’s exact test. We calculated 95% CIs for all categorical outcomes using the Clopper-Pearson method. Correlations between the two immunological endpoints were determined using Cox regression models. Correlations between CD4 count and antibody levels was examined by Spearman’s rho test. Correlations between the number and proportion of participants with adverse reactions or events and the detailed safety profiles were compared across groups by Chi-square test or Fisher’s exact test. Analyses were conducted using SPSS software, version 25.0 (IBM SPSS Inc). A two-sided p < 0.05 was considered statistically significant.

## Results

### Characteristics of the enrolled individuals

We recruited 48 PLWH plus 38 HNC between March to June 2021. All the participants were vaccinated and finished a 70-day follow-up, during which two PLWH participants and two HNCs were lost to follow-up. The median (IQR) age of PLWH was 36 (31–42) years and 87% were males. All PLWH were on ART, 89% had virus suppressed (41/46), and the median CD4 count of PLWH was 523 cells/µL. The age of HNC was similar (median 31, IQD 29–39) to PLWH and 50% were males. CD4 counts were > 500 cells/µL in all participants of HNC (Table 1).

**Table 1:** Baseline Characteristics of the Study Participants in Wuhan, China (N = 84), 2021

Characteristics	PLWH group	HNCs group
No. of participants	46	38
Age in years, median (IQR)	36 (31–42)	31 (27–39)
Men, No. (%)	40 (87)	19 (50)
Duration of infection, years	6 (3, 8)	–
Antiretroviral therapy, No. (%)	46 (100)	–
HIV-VL < 50 copies/mL, No. (%)	41 (89)	–
CD4 count (cells/µL), median CD4 count < 200, No. (%) CD4 count 200–349, No. (%) CD4 count 350–499, No. (%) CD4 count > 500, No. (%)	523 (351, 653) 2 (4) 8 (17) 11 (24) 25 (55)	675 (540, 828) 0 (0) 0 (0) 0 (0) 38 (100)

Data are n (%), Standard Deviation (SD) or median (IQR). Data are for participants with HIV and without HIV included in this analysis

### Safety Outcomes

The adverse events are shown in Table 2. Within 7 days after the first dose, adverse reactions were reported by 14 (30%) and 12 (32%) PLWH and HNC, respectively. The most common adverse reactions after the first dose among PLWH were injection site pain (11/46, 24%), followed by fatigue (4/46, 9%). Fatigue was less common among HIV-negative participants than PLWH (Table 2). Among both PLWH and HNC, there were no increases in reported systemic and local reactions after receiving the second dose. Injection site pain (4/46, 9%) and fatigue (1/46, 2%) were the most reported adverse reactions after the second dose, occurring among PLWH (Table 2). These events were similar compared to those among HNC (Table 2). Adverse reactions in both groups were mild, transient, self-limiting, and did not require any treatment. None of the unsolicited adverse events were observed in both PLWH and HNC.

**Table 2:** Adverse reactions within 7 days after the first and second dose of vaccination

Adverse reaction	PLWH group (n = 46)		Control group (n = 38)		P value
First dose					
Total adverse reactions	14 (30.4)		12 (31.6)		0.91
Systemic reactions	5 (10.9)		2 (5.3)		0.35
Coughing	1 (2.2)		2 (5.3)		0.45
Diarrhea	0		0		–
Fatigue	4 (8.7)		0		0.06
Fever	0		0		
Headache	0		0		–
Nausea and vomiting	0		0		–
Pruritus (non-inoculated site)	0		0		–
Local reactions	14 (30.4)		11 (28.9)		0.88
Itching	0		0		-
Pain	11 (23.9)		9 (23.7)		0.98
Redness	1 (2.2)		1 (2.6)		0.89
Swelling	2 (4.3)		1 (2.6)		0.67
Rash	0		0		–
Second dose					
Total adverse reactions	5 (10.9)	9 (23.7)		0.12	
Systemic reactions	1 (2.2)	3 (7.9)		0.22	
Coughing	0	2 (5.3)		0.12	
Diarrhea	0	0		–	
Fatigue	1 (2.2)	1 (2.6)		0.89	
Fever	0	0			
Headache	0	0		–	
Nausea and vomiting	0	0		–	
Pruritus (non-inoculated site)	0	0		–	
Local reactions	4 (8.7)	6 (13.8)		0.32	
Itching	0	0		-	
Pain	4 (8.7)	5 (13.2)		0.51	
Redness	0	1 (2.6)		0.27	
Swelling	0	0		–	
Rash	0	0		–	

Data are shown as No. of participants with the event (%). A participant was only counted once in the specific reaction category even though a participant could have more than 1 adverse reaction. P value calculated using Chi-square test or Fisher’s exact test

## Neutralizing antibody responses to vaccination

**Fig. 1:**
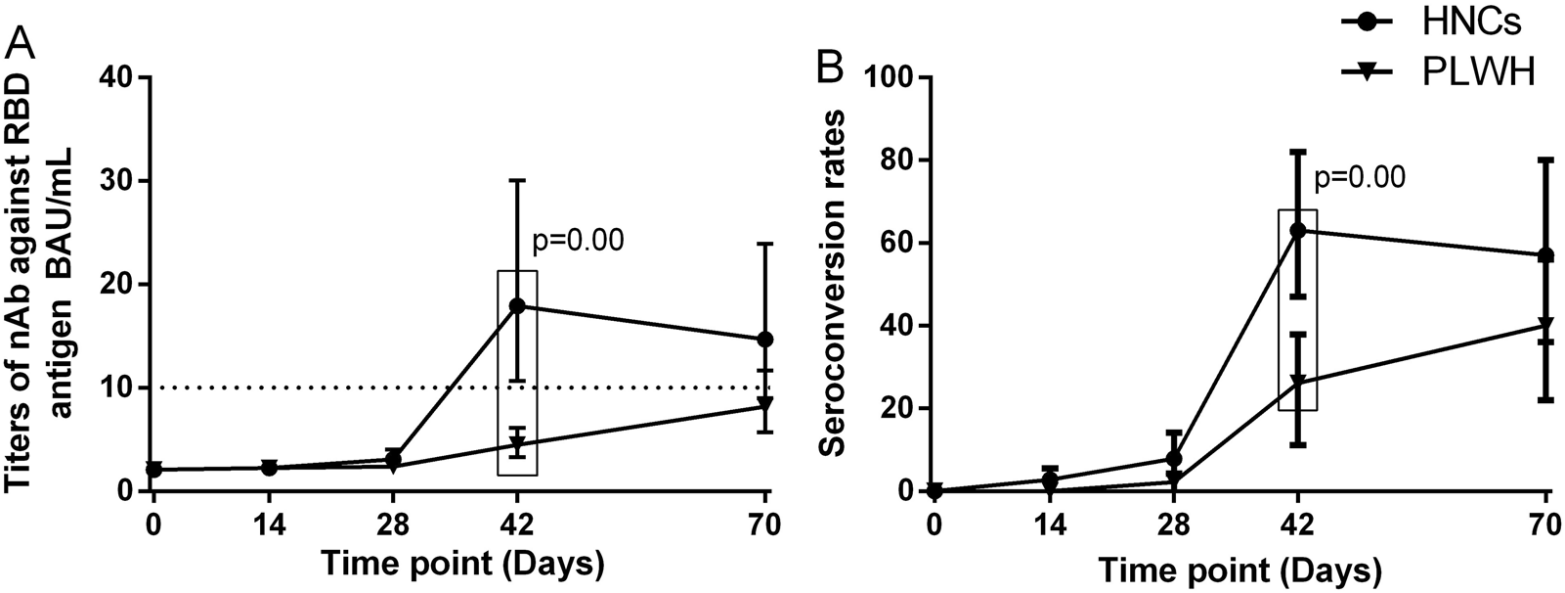
Neutralizing antibody responses to vaccination with inactivated COVID-19 vaccine among PLWH and HNCs. Titers (**A**) and seroconversion rates (**B**) of Neutralizing antibody against RBD antigen at days 0, 14, 28, 42 and 70 after vaccination. The threshold for a positive response is shown by the hashed line at 10 BAU/mL. Data points are medians (error bars represent 95% CI). P value calculated using Mann–Whitney U test (**A**) or Fisher’s exact test (**B**)

None of the participants had any detectable nAbs at baseline. Forty-two days after the first dose of vaccination, GMC and seroconversion rate of nAbs among PLWH were 4.46 BAU/mL (95% CI 3.18–5.87) and 26% (95% CI 14–41), which were both significantly lower than that among HNC [GMC (18.28 BAU/mL, 95% CI 10.33–32.33), seroconversion rate (63%, 95% CI 44–79)] (Fig. 1). At day 70, the GMC and seroconversion rate of nAbs among PLWH [GMC (8.07 BAU/mL, 95% CI 5.67–11.48), seroconversion rate (39%, 95% CI 24–58)] were slightly lower than that among HNC [GMC (11.09 BAU/mL, 95% CI 6.68–18.42), seroconversion rate (57%, 95% CI 34–78)] (Fig. 1), even not significant. The GMC among PLWH at day 42 was significantly lower than that among HNC at day 70 (p = 0.03), while the seroconversion rate of the two groups was similar (p = 0.07).

## Binding-antibody responses to vaccination

**Fig. 2:**
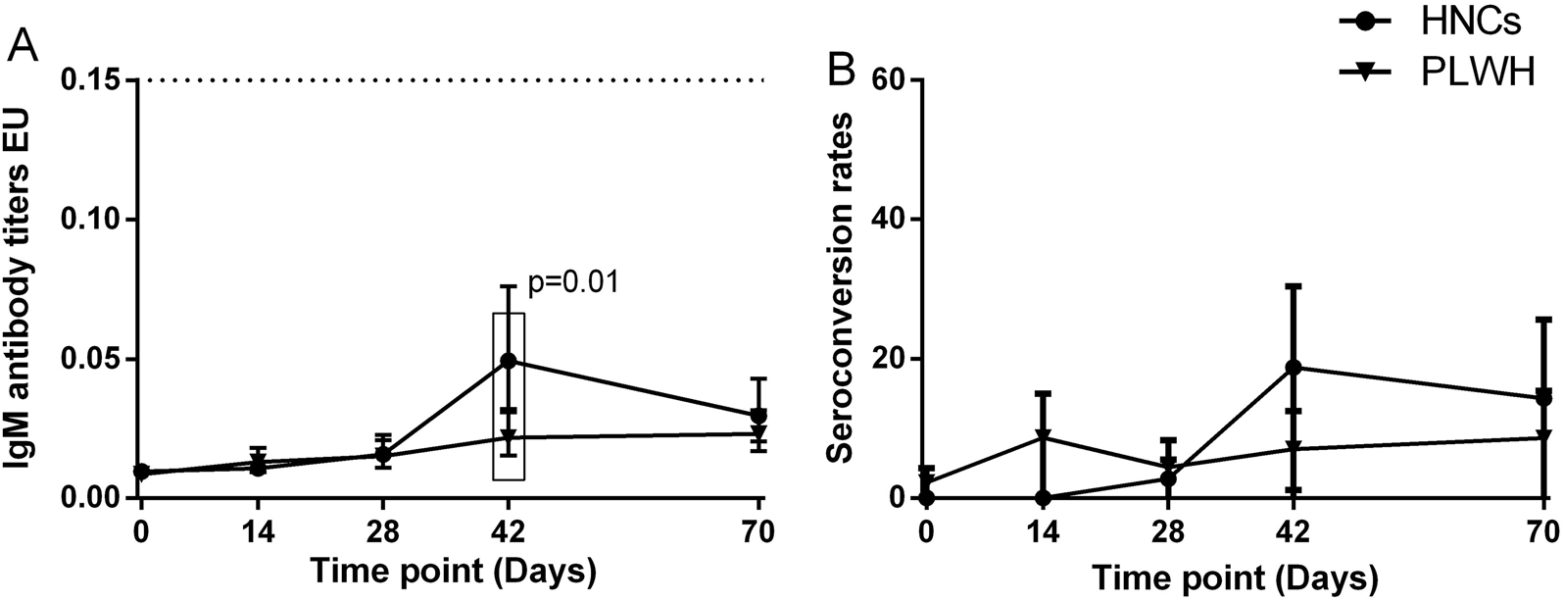
IgM antibody responses to vaccination with inactivated COVID-19 vaccine in PLWH and HNCs. Titers (**A**) and seroconversion rates (**B**) of IgM at days 0, 14, 28, 42 and 70 after vaccination. The threshold for a positive response is shown by the hashed line at 0.15 EU/mL. Data points are medians (error bars represent 95% CI). P value calculated using Mann–Whitney U test (**A**) or Fisher’s exact test (**B**)

**Fig. 3:**
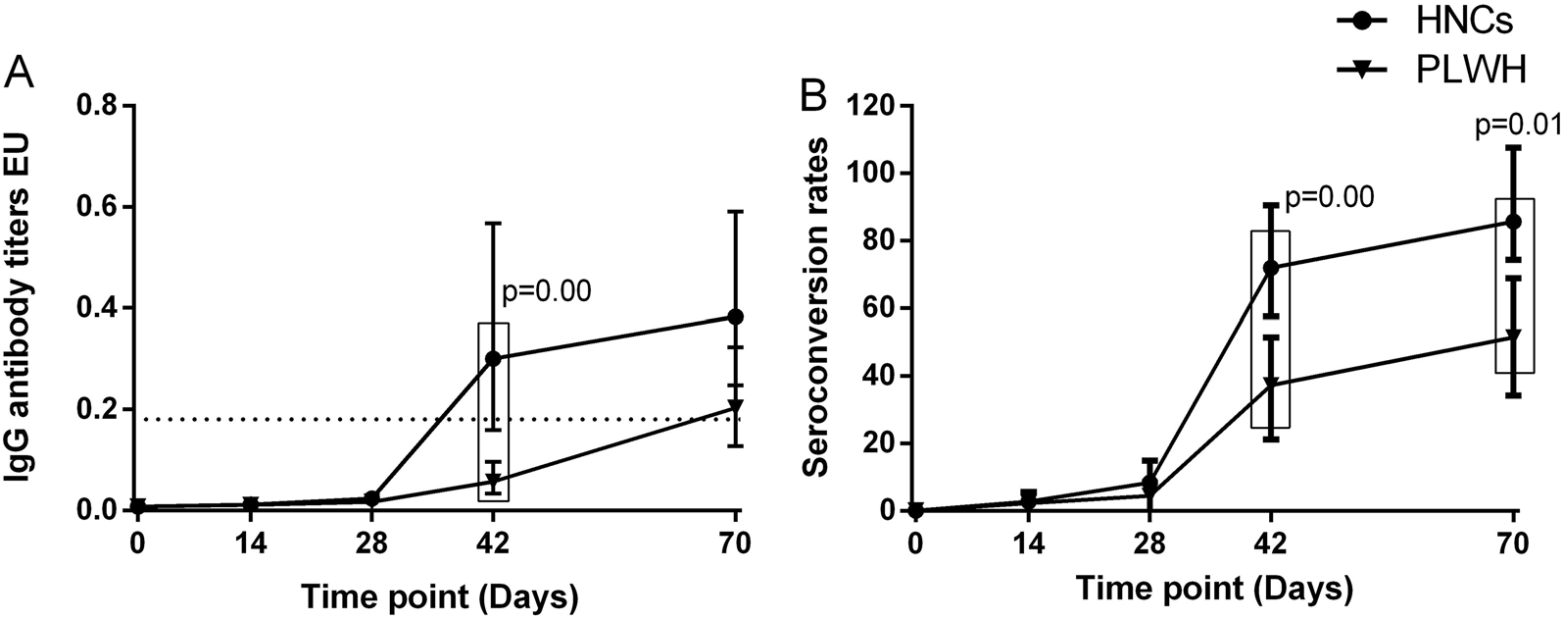
IgG antibody responses to vaccination with inactivated COVID-19 vaccine in PLWH and HNCs. Titers (**A**) and seroconversion rates (**B**) of IgG at days 0, 14, 28, 42 and 70 after vaccination. The threshold for a positive response is shown by the hashed line at 0.18EU/mL. Data points are medians (error bars represent 95% CI). P value calculated using Mann–Whitney U test (**A**) or Fisher’s exact test (**B**)

One participant of PLWH had detectable specific IgM antibodies at baseline. None of the participants had any detectable IgG antibodies at baseline. IgM responses among PLWH peaked at day 70, while among HNC, they peaked at day 42 and sustained at day 70. The geometric mean ELISA units (GMEU) of IgM among PLWH at day 42 was 0.022 BAU/mL (95% CI 0.015–0.031), which was significantly lower than that among HNC [GMC (0.047 BAU/mL, 95% CI 0.029–0.075) (Fig. 2). The seroconversion rate of IgM among PLWH was slightly lower than that among HNC at day 42 but was not significant (Fig. 2). There was no difference in IgM responses between PLWH and HNC in 70 days (Fig. 2). The IgG responses in both PLWH and HNC groups reached their peak at day 70. By day 42, 16/43 (37%) of PLWH showed seroconversion of IgG, increasing to 18/35 (51%) by day 70, with GMEU of 0.062 EU (95% CI 0.036–0.106) and 0.193 (95% CI 0.119–0.313), respectively (Fig. 3). GMEU and seroconversion of IgG antibody at day 42 [GMEU (0.315 BAU/mL, 95% CI 0.155–0.635), seroconversion (72%, 95% CI 53–86)] and day 70 [GMEU (0.379 BAU/mL, 95% CI 0.224–0.653), seroconversion (86%, 95% CI 64–97)] among HNC were significantly higher than that among PLWH (Fig. 3).

## Factors associated with seroconversion

The results of Cox Regression analysis indicate that there was a positive correlation between IgG (anti-S and anti-N) and nAbs at day 70 in both study groups. However, age, gender, comorbidities, and CD4 counts were not associate with the nAbs and IgG seroconversion at day 70 among PLWH (Additional file 1: Table S1). No significant correlation between CD4 count and nAbs/IgG levels was observed in PLWH at day 42 (nAbs Spearman’s ρ = − 0.14, P = 0.36, IgG ρ = − .04, P = 0.79) and day 70 (nAbs Spearman’s ρ = 0.19, P = 0.27, IgG ρ = 0.01, P = 0.97). The seroconversion of qualitative IgM and IgG antibodies was consistent with those of semi-quantitative results.

## Discussion

Understanding the immune response to the COVID-19 vaccine is essential in preparing additional measures for preventing SARS-CoV-2 infection. This prospective study reports on the safety and immunogenicity of an inactivated COVID-19 vaccine in PLWH and HNC. Our study extends the existing literature by reporting the immune response to COVID-19 vaccination among PLWH, comparing it with HNC, and evaluating the vaccine safety among both groups. This integrative analysis demonstrates that the early humoral immune response to inactivated COVID-19 vaccine is delayed and weaker among PLWH than in HNC.

Studies have shown that varied COVID-19 vaccines currently used are safe in adults [6, 7, 19]. However, there is a particular concern for those with primary or secondary immunodeficiencies, who are at an increased risk of SARS-CoV-2 infection or COVID-19 mortality and may have less responsiveness to vaccination. In this study, the adverse reactions of our participants (all of whom were on ART and majority had virus suppressed and CD4 count > 350 cells/µl) were often mild and moderate in severity and self-limiting. The incidence rate of adverse events among PLWH was similar to the HNC in our study but lower than the results of other types of vaccines, for example, the mRNA COVID-19 vaccine in America among healthy adults or the ChAdOx1 nCoV-19 vaccine in PLWH and without HIV in UK [7, 13]. Therefore, inactivated COVID-19 vaccination for PLWH is relatively safe. However, these comparisons should be interpreted cautiously due to the small sample size in the studies.

We found that nAbs responses to inactivated COVID-19 vaccine were delayed and weaker in PLWH compared to HNC. For example, NAbs responses among PLWH at day 42 were significantly lower than that among HNC. Consistently, the findings of our previous study showed that the IgG positive conversion rate for SARS-CoV-2 is relatively lower and quickly lost in PLWH infected with SARS-CoV-2[20]. However, these findings are different from findings of other studies conducted in South Africa and UK, which suggested that the serological responses produced by the COVID-19 vaccine among PLWH are similar to those among HNC [12, 13]. A study about the ChAdOx1 nCoV-19 vaccine showed that there was no statistical difference in magnitude or seroconversion of SARS-CoV-2 spike-specific humoral or cellular responses among PLWH and HNC [13]. It is possible PLWH with viral suppression may have impaired antigen-specific B-cells and T-cells response [21]. Additionally, HIV infection is assumed to be associated with impaired antibody responses to other vaccines such as influenza vaccine [16, 22]. Epidemiological studies have reported that nAbs titers vary widely in convalescent serum samples and may be related to several factors (like age, sex, disease severity, and days since infection) [23, 24].

Virus-specific IgG responses in both groups began to be produced between day 28 and day 42 and boosted between day 42 and 70 after being given the first dose of vaccine in our study. Notably, the IgG responses of PLWH were significantly lower than those of HNC at both 42 and 70 days. While the exact duration of immunity conferred by COVID-19 vaccine remains unresolved, induction of nAbs and presence of antibodies to SARS-CoV-2 is thought to be associated with of protection against SARS-CoV-2[6, 19]. Other studies on antibody response to mRNA COVID-19 vaccination have demonstrated that CD4 counts < 200 cells/µl developed relatively lower antibody levels [25]. Moreover, CD4 count is purportedly associated with decreased humoral response to multiple vaccines, including hepatitis A, hepatitis B, and pneumococcus vaccines among PWLH [26–28]. That could likely be due to the role of CD4 cells in germinal center formation [21]. However, our study found no correlation between antibodies responses and CD4 counts. Whether vaccine-induced antibody levels could persist as the exact duration of immunity conferred by the COVID-19 vaccine remains unresolved. Therefore, a long-term study is needed to determine the difference in duration of nAbs among PLWH and HNC.

This preliminary prospective study has several limitations. First, the sample size was small, and we only followed up with participants for 70 days after vaccination. That makes it impossible to generalize our findings for now. We will continue to enroll more participants and further follow up with the existing participants, to determine the long-term immune responses among PLWH. Secondly, there was an imbalance in the sex distribution of PLWH. Nonetheless, a previous study found equivalent responses in males and females using this vaccine, and this may mitigate some of the sex imbalance in this study [8]. Thirdly, our cohort was predominantly PLWH with high CD4 + T cell counts and long-term access to ART. Therefore, more data from PLWH with CD4 + T cell count below 350 cells/µl or without ART is required. Lastly, this study focused on responses to the inactivated COVID-19 vaccine. So it is impossible to comment on potential responses that PWLH might make to other SARS-CoV-2 vaccines or other vaccines interval durations.

## Conclusions

This is the first report of the immune response of inactivated COVID-19 vaccine (WIBP-CorV) among PLWH. In this study of PLWH, vaccination with inactivated COVID-19 vaccine was well tolerated. Early humoral immune response to the inactivated COVID-19 vaccine was weaker and delayed among the PLWH population than that among HNC. This observation remained consistent regardless of a high CD4 count with effective antiretroviral therapy.

## Supplementary Information

**Additional file 1: Table S1**. The antibody seroconversion and associated factors among PLWH sub-group.

## Data Availability

The datasets used and/or analyzed during the current study are available from the corresponding author on reasonable request.
