# Correction: Immune response and safety to inactivated COVID-19 vaccine: a comparison between people living with HIV and HIV-naive individuals

**DOI:** 10.1186/s12981-024-00655-y

**Published:** 2024-09-23

**Authors:** Shi Zou, Mengmeng Wu, Fangzhao Ming, Songjie Wu, Wei Guo, Gifty Marley, Zhongyuan Xing, Zhiyue Zhang, Minxia Zeng, Chao Sun, Jianfeng Zhang, Weiming Tang, Ke Liang

**Affiliations:** 1 Department of Infectious Diseases, Zhongnan Hospital of Wuhan University, Wuhan, China; 2 Wuchang District Center for Disease Control and Prevention, Wuhan, China; 3 Department of Nosocomial Infection Management, Zhongnan Hospital of Wuhan University, Wuhan, China; 4 Department of Pathology, Zhongnan Hospital of Wuhan University, Wuhan, China; 5 Department of Pathology, School of Basic Medical Sciences, Wuhan University, Wuhan, China; 6 School of Public Health, Nanjing Medical University, Nanjing, China; 7 Wuhan University School of Basic Medical Sciences, Wuhan, China; 8 Zhuhai Livzon Diagnostics CO, LTD, Zuhai, China; 9 Guangdong Second Provincial General Hospital, Guangzhou, China; 10 The University of North Carolina at Chapel Hill Project-China, Guangzhou, China; 11 Wuhan Research Center for Infectious Diseases and Cancer, Chinese Academy of Medical Sciences, Wuhan, China; 12 Hubei Engineering Center for Infectious Disease Prevention, Control and Treatment, Wuhan, China; 13 Dermatology Hospital, Southern Medical University, Guangzhou, 510095 China

**Correction to: AIDS Research and Therapy (2022) 19:33**

10.1186/s12981-022-00459-y

In this article [1], the affiliation details for Author Weiming Tang were incorrectly given as “Guangdong No. 2 Provincial People’s Hospital, University of North Carolina at Chapel Hill Project-China, Guangzhou 510095, China” but should have been “Guangdong Second Provincial General Hospital, Guangzhou, China”.
