# Supplementary material for: Immune response and safety to inactivated COVID-19 vaccine: a comparison between people living with HIV and HIV-naive individuals
Source: AIDS Res Ther. 2022 Jul 5;19:33. doi: 10.1186/s12981-022-00459-y (PMC9253234; doi:10.1186/s12981-022-00459-y)
Supplement: Supplementary file 1 — Additional file 1: Table S1. The antibody seroconversion and associated factors among PLWH sub-group. [file 12981_2022_459_MOESM1_ESM.docx]

**Supplement:**

Table 1. The antibody seroconversion and associated factors among PLWH sub-group

| Variables | nAbs (AOR and 95% CI) | IgG (AOR and 95% CI) |
| --- | --- | --- |
| Age | 1.33 (0.45-3.99) | 1.44 (0.55-3.81) |
| Male | - | 0.33 (0.04-2.43) |
| Comorbidities | - | 0.87 (0.11-6.69) |
| CD4 count (cells/μL) | 1.22 (0.27-5.63) | 0.44 (0.15-1.41) |
